# Isolation of *Burkholderia pseudomallei* from a Pet Green Iguana, Belgium

**DOI:** 10.3201/eid2412.171661

**Published:** 2018-12

**Authors:** Tom Hellebuyck, Pierre Wattiau, Filip Boyen, Ilse Moeremans, Nancy H. Roosens, Kevin Vanneste, An Garmyn, Veronique Saey, Frank Pasmans, Freddy Haesebrouck

**Affiliations:** Ghent University, Merelbeke, Belgium (T. Hellebuyck, F. Boyen, I. Moeremans, A. Garmyn, V. Saey, F. Pasmans, F. Haesebrouck);; Veterinary and Agrochemical Research Centre, Brussels, Belgium (P. Wattiau);; Sciensano, Ixelles, Belgium (N.H. Roosens, K. Vanneste)

**Keywords:** Burkholderia pseudomallei, bacteria, Iguana, green iguana, reptiles, granulomatous hepatitis, melioidosis, reservoirs, zoonoses, Belgium

## Abstract

We isolated *Burkholderia pseudomallei*, the causative agent of melioidosis, from liver granulomas of a pet green iguana (*Iguana iguana*) in Belgium. This case highlights a risk for imported green iguanas acting as a reservoir for introduction of this high-threat, zoonotic pathogen into nonendemic regions.

The highly pathogenic, gram-negative bacterium *Burkholderia pseudomallei* is the causative agent of melioidosis, which is endemic to countries in Southeast Asia and in northern Australia and an emerging infectious disease in several tropical developing countries (*1**,**2*). Human cases in Europe are limited to patients who traveled to disease-endemic regions. In Belgium, the last case was documented in 2013 in a 44-year-old man who had traveled to Madagascar (*3*). *B. pseudomallei* is classified as a tier 1 overlap select agent by the US Federal Select Agent Program (*4*). Postexposure prophylaxis and postexposure monitoring should be planned for persons who have had high-risk exposures, such as certain laboratory procedures with the organism that were not conducted under Biosafety Level 3 conditions (*5*).

Infected humans and importing of infected animals can introduce melioidosis into nonendemic areas (*2**,**6*). However, importing of infected animals has not yet been associated with epizootic transmission. Infection commonly occurs through cutaneous inoculation, ingestion of contaminated soil or water, or inhalation of aerosolized bacteria (*7**,**8*). Although the incubation period in humans is typically 1–21 days, clinical disease might develop years after infection (*9*). The incubation period in naturally infected animals is not known (*10*).

Acute melioidosis results predominantly in pneumonia and septicemia. Chronic infection is associated with abscesses of the liver, lungs, spleen, and skin (*9*). Because of the intrinsic resistance of the bacterium to many antimicrobial drugs, combined with the inability to provide appropriate medical care in disease-endemic developing countries, overall case-fatality rates might exceed 70% (*1*,*4*,*9*). *B. pseudomallei* is rarely reported in animals other than cattle, goats, and swine (*10*).

In reptiles, isolation of *B. pseudomallei* has been anecdotally documented in crocodiles (*10*), and clinical infections have been reported in 2 pet green iguanas in California, USA (*11*) and a pet green iguana in Prague (Czech Republic) (*4*). Dermal abscesses were observed in the iguana from the Czech Republic and in 1 of the iguanas from California, and hepatic masses were observed in the second iguana from California.

Because of the variable clinical manifestations of melioidosis and limited value of conventional bacterial methods for identification of *B. pseudomallei*, diagnosis of melioidosis can be challenging (*2**,**11*). If one considers the highly pathogenic and zoonotic nature of *B. pseudomallei*, use of appropriate molecular detection methods is crucial to warrant correct identification and discrimination of *B. pseudomallei* from other *Burkholderia* species (*11*). Next-generation sequencing might be a valuable supplement to current identification and diagnostic methods.

## The Study

A 5-year-old female green iguana (*Iguana iguana*) showed acute onset of lethargy, anorexia, and general weakness. The iguana had been purchased 4.5 years earlier by private owners from a pet shop in the Netherlands that imported the iguana from a captive breeding operation in Central America. Serum biochemical and hematologic tests showed hyperuricemia, hyperphosphatemia, an increased level of aspartate aminotransferase, hyperglobulinemia, nonregenerative anemia, and severe leukocytosis in comparison with physiologic reference ranges for these conditions (*12*). Ultrasonography showed hepatomegaly, multiple hyperechoic hepatic masses, and severely enlarged, hyperechoic kidneys. A presumptive diagnosis of hepatitis, kidney failure, and septicemia was made. Because the iguana did not respond to supportive treatment and its general condition continued to deteriorate, the owners agreed to euthanize the iguana on the fifth day after signs began.

During necropsy, pronounced renomegaly and hepatomegaly, as well as granulomatous hepatitis was observed. Histologic evaluation of hematoxylin and eosin–stained sections of liver showed coalescent granulomas throughout the liver parenchyma, with central necrosis surrounded by activated macrophages and giant cells (Figure). Gram, periodic acid–Schiff, and acid-fast staining did not show intralesional gram-positive or acid-fast bacteria or fungi. Microbiological examination of liver tissue yielded a pure and abundant culture of small colonies of gram-negative rods that could not be identified by using standard biochemical identification tests.

**Figure:**
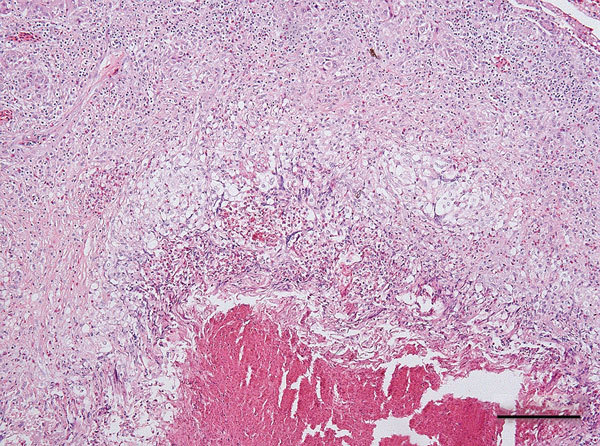
Coalescent granuloma in liver parenchyma of a pet green iguana (*Iguana iguana*) infected with *Burkholderia pseudomallei*, Belgium. Hematoxylin and eosin stain shows central necrosis surrounded by activated macrophages and giant cells. Scale bar indicates 200 μm.

Sequencing of the 16S rRNA gene of the isolate showed 100% identity with *B. pseudomallei* (1,382 bp). Sequence data were deposited in GenBank under accession no. MF523223. After culturing of the isolate under Biosafety Level 3 conditions, we performed multilocus sequence typing–derived PCR and real-time PCR as described by Wattiau et al. (*13*), which confirmed identification of the isolate as *B. pseudomallei*.

We used next-generation sequencing to obtain the genome sequence of the isolate, which was deposited in the Short Read Archive under accession no. SRR6056996. The genome sequence was used to infer its multilocus sequence type (ST) as ST518. Isolate information was deposited accordingly in the *B. pseudomallei* database (https://pubMLST.org) under ID 5121 (*14*). Of 5,600 deposited isolates that we accessed on June 18, 2018, ST518 matched only with *B. pseudomallei* isolates (ID 3330) obtained from a human who was infected in Costa Rica in 2009 (ID 1928) (*14*) and with 2 isolates obtained from abscesses in pet iguanas in 2007 (ID 5008) and 2012 (ID 3330) (*11*). We found that ID 3330 differed from the isolates from Central America by only 3 single-nucleotide polymorphisms, which strongly suggested that the iguanas were infected in Central America before transport abroad (*15*). However, whether captive iguanas might acquire infections through contact with other imported animals (e.g., in pet shops or in captive collections in nonendemic countries) cannot be excluded.

After we identified of the isolate as *B. pseudomallei*, we collected 100 mL of water from a plastic water bath and a swab specimen from the emptied water reservoir from the housing of the iguana and tested these samples by using real-time PCR. All samples showed negative results.

The zoonotic potential of the bacterium was discussed with the owners and personnel who came into contact with the iguana or samples that were collected from the lizard. Although the owners did not have a clinical history that could indicate *B. pseudomallei* infection, they were advised to consult with their physicians about potential exposure. Although the owners refused postexposure prophylaxis and postexposure monitoring, postexposure prophylaxis was given to a selected number of staff members as recommended by Lipsitz et al. (*5*) because of potential exposure to the pathogen.

## Conclusions

Previously reported *B. pseudomallei*–infected iguanas (*4**,**11*) and the 1 reported in this study were presumably imported from disease-endemic regions, highlighting the potential role of this species as a reservoir of *B. pseudomallei*. On the basis of the relatedness of ST518 with an isolate obtained from a human in Costa Rica (*14*) and with 2 isolates from iguanas that presumably became infected in Central America (*11*), whose genomes were later confirmed to be closely related (*15*), our findings support the hypothesis that the iguana we report became infected in the captive breeding facility in Central America before importation to Europe.

If one considers the long incubation period observed for green iguanas, these reptiles might shed the bacterium unnoticed for years before nonspecific chronic disease develops. Transmission from infected captive iguanas to humans might occur through contact with stool, infected tissue, or biting and scratching lesions. Indirect infection might originate from wound infection through contaminated soil or water or inhalation of aerosolized bacteria.

In conclusion, traders, (para)veterinarians, and laboratory staff members who handle green iguanas or samples obtained from this species are susceptible to infection. In addition, physicians who are consulted by pet green iguana owners should be aware that these animals and their environment could potentially harbor *B. pseudomallei*.
